# Understanding Alzheimer’s Disease Knowledge in Low-Income, Racially Diverse Older Adults

**DOI:** 10.1093/geroni/igaa057.918

**Published:** 2020-12-16

**Authors:** Faika Zanjani, Taylor Wilkerson, Annie Rhodes, Jennifer Inker, Joann Richardson

**Affiliations:** Virginia Commonwealth University, Richmond, Virginia, United States

## Abstract

Individuals demonstrate varying levels of Alzheimer’s disease (AD) knowledge, as well as commonly held misconceptions about the risk factors and nature of the disease. Older adults often demonstrate low scores on AD knowledge scales and African Americans are often specifically not aware of their higher AD risk status compared to other racial groups. We measured the Alzheimer’s knowledge in 60+ community-dwelling adults, as part of a larger study on AD health coaching. Participants (n=20) were recruited from low-income communities within the Richmond, Virginia area. The study sample was 85% African American (n=17) and 55% male (n=11). Participants completed a behavioral psychosocial test battery, including the Alzheimer’s Disease Knowledge Scale. Similar to previous research, this sample of older adults held common misconceptions about AD, including the ideas that mental exercise can prevent AD (80% answered incorrectly) and individuals with AD are incapable of making decisions about their care (70% answered incorrectly). In this sample, the majority of African American older adults were aware of the fact that they have the highest risk for developing AD (20% answered incorrectly) compared to other racial groups. Analyses found no significant relationship between AD knowledge and health outcomes, alcohol consumption, or education. In conclusion to reduce AD risk, addressing AD knowledge in minority low-income population is important and needed. This is especially relevant since African American older adults are more likely to live in communities rather than nursing or assisted living facilities, receiving less access to interventions and research innovation.

